# Relationships Among Leaders' and Followers' Work Engagement and Followers' Subjective Career Success: A Multilevel Approach

**DOI:** 10.3389/fpsyg.2021.634350

**Published:** 2021-03-11

**Authors:** Qishan Chen, Shuting Yang, Jiayu Deng, Liuying Lu, Jingyi He

**Affiliations:** ^1^Key Laboratory of Brain, Cognition and Education Sciences, Ministry of Education, Guangzhou, China; ^2^Guangdong Key Laboratory of Mental Health and Cognitive Science, Center for Studies of Psychological Application, School of Psychology, South China Normal University, Guangzhou, China; ^3^Beijing Key Laboratory of Applied Experimental Psychology, Faculty of Psychology, National Demonstration Center for Experimental Psychology Education (Beijing Normal University), Beijing Normal University, Beijing, China

**Keywords:** work engagement, subjective career success, work team, leader, follower, multilevel model

## Abstract

Using a sample of 52 work teams (52 work team leaders and their 348 followers) in China, we investigated the influence mechanism of leaders' work engagement on their followers' work engagement and subjective career success. A multilevel structural equation model (MSEM) was applied to analyze the survey data. The results of this study indicated that leaders' work engagement positively influenced their followers' subjective career success, and this relationship was mediated by the followers' work engagement. Implications of these findings, limitations, and directions for future research are discussed in the final section of the paper.

## Introduction

Career success is the goal eagerly pursued by every employee. As it plays an important role in individual lives, organizations, and even society, the past decades have shown a dramatic increase in research interest in career success in both theory and practice (Arthur et al., 2005; Heslin, 2005; Ng et al., 2005; Ng and Feldman, 2014; Hirschi et al., 2018; Heslin et al., 2019; Smale, 2019; Spurk et al., 2019).

In recent years, the research on career success has received growing attention in the fields of psychology and organizational science, especially in studies that have focused on its impact factors (Ramaswami et al., 2010, 2016; De Vos et al., 2011; Stumpf and Tymon Jr, 2012; Spurk and Abele, 2014; Zacher, 2014; Deng et al., 2015; Hirschi and Jaensch, 2015; Akkermans and Tims, 2017; Cenciotti et al., 2017; Hirschi et al., 2018; Suutari et al., 2018). A meta-analysis revealed four categories of predictors of objective and subjective career success: human capital, organizational sponsorship, sociodemographic status, and stable individual differences (Ng et al., 2005; Spurk et al., 2019). With regard to subjective career success, Ng and Feldman (2014) found that it was significantly related to dispositional traits (e.g., emotional stability), social networks (e.g., supervisor support), organizational and job support (e.g., job insecurity), and motivation (e.g., work engagement).

Work engagement is currently one of the most studied topics in organizational science, and it is closely tied to employees' career success. Considerable evidence has proven the impact of work engagement on career success. Ng et al.'s (2005) meta-analysis and Breevaart et al.'s (2015) work indicated that work engagement can predict objective career success and that it was positively correlated with subjective career success. Although many studies have been conducted on the relationship between work engagement and career success, the existing studies have tended to investigate this relationship from an individual perspective (individual level) rather than from a work team perspective (multilevel).

The main purpose of the present research is to examine the influence of team leaders' work engagement on their followers' subjective career success and the multilevel mediating effect of followers' work engagement from a multilevel perspective.

### Subjective Career Success

Career success is defined as an accumulated positive psychological accomplishment, and work outcomes resulting from individual work experiences encompass both objective and subjective criteria (Arthur et al., 2005; Pan and Zhou, 2015; Shockley et al., 2016). Researchers distinguish subjective career success from objective career success that can be directly observed, measured, and verified by an impartial third party, while subjective career success is defined as the focal career actor's evaluation and experience of achieving personally meaningful career outcomes including that actor's internal apprehension, perceptual evaluation, and sense of his or her own career success (Judge et al., 2010; Hogan et al., 2013; Shockley et al., 2016).

A research found that an increasing number of employees define their career success in terms of subjective indicators, such as job satisfaction or career satisfaction, rather than in terms of objective indicators, such as salary and promotion (Ng and Feldman, 2014). Although objective and subjective career successes are considered to be positively related, the two constructs are empirically distinct. Career success cannot be predicted solely through salary or promotion. In contrast, some people may consider their career success in terms of job satisfaction.

The antecedents of subjective career success have been extensively researched. The research has found that marital status, educational level, and degree type can predict objective success. In contrast, motivational and organizational variables have been found to explain significant amounts of variance in subjective success (Mohd Rasdi et al., 2011). A meta-analysis of 216 independent samples, representing a total of 12,567 employees, showed that career hurdles, job insecurity, low emotional stability, low supervisor support, and low work engagement were all significantly related to lower subjective career success (Ng and Feldman, 2014).

Employees' subjective career success is especially influenced by their perceptions and reactions to their current jobs and organizations (Ng et al., 2005; Ng and Feldman, 2014; Baethge et al., 2017; Moon and Choi, 2017; Peng et al., 2019). Therefore, particular attention must be paid to the issue of work engagement in relation to job perception. In addition, the variables at the team level have a significant impact on employees' subjective career success. Hence, we must also pay more attention to how leaders' character influences followers' subjective career success.

### From Leaders' Work Engagement to Followers' Work Engagement

Work engagement is a fulfilling, positive work-related state of mind that is characterized by vigor, dedication, and absorption (Bakker et al., 2011; Vigoda-Gadot et al., 2013). Vigor refers to high levels of energy and mental resilience in working. Dedication refers to being strongly involved and experiencing a sense of significance, enthusiasm, and challenge in one's work. Absorption is characterized by being fully concentrated and happily engrossed in work such that time passes quickly (Bakker and Leiter, 2010; Schaufeli and Bakker, 2010; Bakker, 2011).

A work team consists of team members who have the same commitment and responsibility to achieve a particular goal or to accomplish a task. Because a work team operates in an organizational context, information communication and interpersonal interactions occur between team leaders and followers as well as among followers (Hart and Mcleod, 2003; Kozlowski and Bell, 2003; Loi et al., 2017).

The team leader not only instructs and supervises the followers but also offers them information and resources in their daily work. Followers accept orders from the leader and accomplish their assigned tasks. During this process, the leader's psychological and work state may influence the followers' work attitude and behavior in formal and informal ways.

Work engagement is conceptualized as a state with important characteristics such as openness to development and the existence of contagion effects. In other words, the work engagement in the leader–follower relationship and the work engagement in the follower–follower relationship could easily impact one another (Bakker and Xanthopoulou, 2009; Schaufeli et al., 2009; Zhu et al., 2009; Zettler and Hilbig, 2010; Gutermann et al., 2017; Lu et al., 2018).

Such influence varies with leaders. Leaders who are more engaged in work tend to be hopeful, motivated to succeed, and likely to establish challenging goals. They are willing to explore ways to solve problems. In other words, they have positive expectations of the work environment, resulting in a positive attitude and strong job performance. Leaders' with such characteristics and behavior will easily affect their followers and cause them to experience more positive emotions, which may accordingly improve their work engagement (Johnson, 2009; Tims et al., 2011; Gutermann et al., 2017). Conversely, it is difficult to imagine how team members might be inspired and encouraged by an unengaged leader.

Moreover, leaders are always regarded as examples for their followers to imitate. In this way, leaders become an influential source of information for their followers regarding appropriate attitudes and behaviors. If leaders display high levels of work engagement, their team will be willing to observe and perceive the positive work results of their leaders' work engagement. It would be beneficial for team members to develop positive and optimistic job expectations as well as a stronger motivation for success, which could increase their work engagement (Schaufeli et al., 2009; Ambrose et al., 2013; Lehmann-Willenbrock et al., 2015).

Therefore, in this research, we propose the following hypothesis:

Hypothesis 1: Leaders' work engagement positively relates to their followers' work engagement.

### Followers' Work Engagement and Subjective Career Success

In recent years, a large number of studies in the fields of psychology and management have shown that work engagement contributes to desired job outcomes for both individuals and organizations (Shuck and Herd, 2012; Carasco-Saul et al., 2015). As a positive work-relevant experience and a condition of mind, work engagement is important for individuals in the process of improving themselves and achieving success. According to the job demands–resources (JD-R) model (Bakker and Demerouti, 2008), employees who have job resources and personal resources are confident in their abilities and are optimistic about their future. This allows them to be more engaged in their jobs and to avoid job burnout. Thus, they will remain motivated and able to better cope with work challenges and achieve career success (Xanthopoulou et al., 2007; Akkermans et al., 2013; Chen et al., 2018).

Empirical studies have supported the general belief that work engagement contributes to subjective career success (job satisfaction). For example, Ng and Feldman (2014) and Elams-Atay (2017) found that work engagement was positively correlated to subjective career success. In addition, work engagement was predictive of job performance and satisfaction (Breevaart et al., 2015; Eldor and Harpaz, 2016; Guo et al., 2017; Ngo and Hui, 2017).

Bakker et al. (2008) noted that engaged workers perform better than unengaged workers because they often experience positive emotions, including happiness, joy, and enthusiasm; maintain better psychological and physical health; conserve their own job and personal resources (e.g., support from others); and communicate their engagement to others.

Thus, we expect work engagement comprising vigor, dedication, and absorption to improve follower subjective career success by enhancing an individual's overall motivation and perseverance. We propose the following hypothesis:

Hypothesis 2: Followers' work engagement positively relates to their subjective career success.

An engaged leader plays an important role in the team. Engaged leaders have been found to be more loyal to their organizations and often maintain better psychological and physical health (Bakker et al., 2008; Halbesleben and Wheeler, 2008; Schaufeli et al., 2009). In addition, leaders who are more engaged can communicate their engagement to their followers (Bakker and Xanthopoulou, 2009; Gutermann et al., 2017). Although leaders' work engagement has an important impact, followers' work engagement is also necessary for followers' career success. It is unlikely for a team member to achieve career success without his or her own engagement.

To extend the previous research on work engagement and follow the logic of hypotheses 1 and 2, we regard leaders' work engagement as a more distal predictor of followers' subjective career success. We believe that leaders' work engagement is likely to play a role in followers' subjective career success by enhancing their work engagement. Supporting this argument, Vincent-Höper et al. (2012) reported that employees' work engagement was found to partially mediate the relationship between transformational leadership characteristics and subjective career success. Many studies have also found that employees' work engagement mediates the link between leaders' relational behaviors and employees' job attitude and performance (Shuck and Herd, 2012; Carasco-Saul et al., 2015).

Based on these findings, we expect followers' work engagement to serve as a mediator of the relationship between leaders' work engagement and followers' subjective career success. We therefore propose the following hypothesis:

Hypothesis 3: Followers' work engagement mediates the positive relationship between leaders' work engagement and their followers' subjective career success.

**Figure 1:**
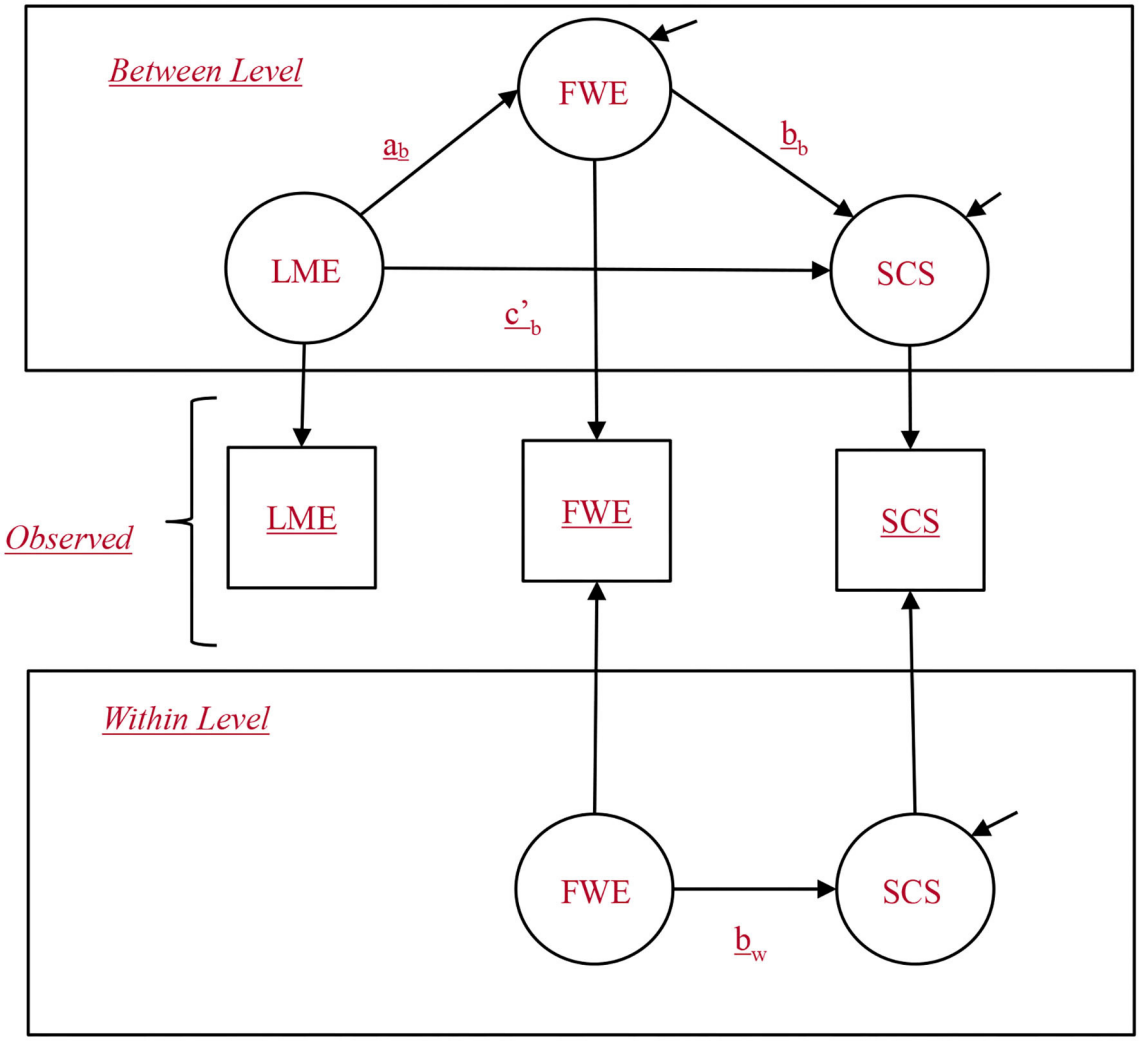
Multilevel structural equation model for the 2-1-1 hypothesized model. SCS, subjective career success; LWE, leaders' work engagement; FWE, followers' work engagement. b subscript represents between level and w subscript represents within level.

To summarize, the hypothesized model is as follows (Figure 1).

## Methods

### Participants

The participants in this research were work teams from 14 public enterprises in Guangdong Province, China. They were similar in terms of their organizational forms, work tasks, work patterns, and performance assessment criteria. A total of 480 questionnaires were distributed in this investigation, and 400 valid questionnaires from both leaders and team members were collected, for an 83.3% participation rate. We collected valid data from a total of 52 work teams, the number of which ranged from 3 to 15, with 7 team members on average. In every work team, each employee has worked with his or her direct supervisor for at least 1 year, with an average of 6.9 years. Of all the participants, 16.4% were males, and 83.6% were females. The age of the participants ranged from 21 to 59 years old, with an average age of 34. The education background of the participants varied from middle school education to master's and above, with 0.3% having completed middle school education and below; 4.9% having completed high school, vocational high school, technical school, or technical secondary school; 43.3% having completed junior college; 51.2% having completed undergraduate education; and 0.3% having completed master's education and above. The length of work of all the participants averaged 12.23 years.

The present research was approved by the research ethics committee of the respective university (referral number: SCNU-PSY-335). All participants provided their written consent before completing the questionnaires. The data were collected and analyzed anonymously.

### Measures

#### Work Engagement

We adopted the Utrecht Work Engagement Scale (UWES) developed by Schaufeli et al. (2002) to measure the participants' work engagement. This 17-item scale contained three dimensions—vigor, dedication, and absorption—with 6, 5, and 6 items for each dimension, respectively. In the present research, we used a 5-point Likert measure (1 = strongly disagree, 2 = disagree, 3 = not sure, 4 = agree, and 5 = strongly agree). The Cronbach's alpha coefficients of leaders' work engagement in vigor, dedication, and absorption were 0.86, 0.89, and 0.85, respectively; those of the followers' work engagement were 0.88, 0.87, and 0.85, respectively. The *ICC*(1) of followers' work engagement was 0.293. Because *ICC*(1) > 0.059, between-group differences existed and could not be neglected (Cohen, 1988).

#### Subjective Career Success

We adopted a 5-item scale, the Career Satisfaction Scale, to measure followers' subjective career success. This one-dimensional scale was developed by Greenhaus et al. (1990) and has been widely used in research. In the present research, we used a 5-point Likert measure (1 = strongly disagree, 2 = disagree, 3 = not sure, 4 = agree, and 5 = strongly agree). The Cronbach's alpha coefficient of the scale was 0.67. The *ICC*(1) of subjective career success was 0.203.

### Measurements and Data Analysis

We sent self-reported questionnaires to both team leaders and followers. The questionnaire for the leaders included a work engagement measurement, while the questionnaire for the followers contained both a subjective career success scale and a work engagement scale. To reduce the participants' concerns and to protect their privacy, all the questionnaires and test compensation were enclosed and sealed in envelopes in advance. On the cover of the questionnaires, the following statement was included: “To protect your privacy, please return the completed questionnaire in the envelope and seal it well.” The participants were assigned a random number for identification and matching.

Regarding the questionnaires for the followers, we adopted Harman's one-factor test to examine common method variance (Malhotra et al., 2006). The goodness-of-fit index of the one-factor model and four-factor model are as follows, respectively: χ^2^ = 888.99, df = 152, RMSEA = 0.118, TLI = 0.78, CFI = 0.80, and SRMR = 0.068 (one-factor); χ^2^ = 517.82, df = 146, RMSEA = 0.086, TLI = 0.88, CFI = 0.90, and SRMR=0.056 (four-factor). The result that the four-factor model fit better than the one-factor model revealed that common method variance was possible in the present research; however, its influence could be small.

SPSS 21.0 was used in this research to perform descriptive statistics, correlation analyses, exploratory factor analysis, and reliability analysis. We used Mplus 7.4 (Muthén and Muthén, 1998-2015) to conduct confirmatory factor analysis and multilevel structural equation modeling (MSEM). Missing data accounted for <5%, and we adopted the EM algorithm to account for it.

## Results

### Descriptive Statistics

The descriptive statistics are shown in Table 1. Leaders' work engagement was positively correlated with followers' work engagement and subjective career success. This was the basis for the next mediation analysis. Followers' educational background was significantly correlated with both followers' work engagement and followers' subjective career success. Thus, followers' educational background was controlled for in our subsequent data analyses.

**Table 1:** Descriptive statistics.

**Variables**	***M***	**SD**	**1**	**2**	**3**	**4**	**5**	**6**	**7**	**8**	**9**
1. Leaders' work engagement	4.03	0.63	–								
2. Sex^a^	0.16	–	–	–							
3. Age	34.84	8.58	–	0.15^**^	–						
4. Educational background	3.46	0.61	–	0.04	−0.38^**^	–					
5. Working years	12.23	9.37	–	0.12^*^	0.95^**^	−0.36^**^	–				
6. Years working in this position	6.88	7.24	–	0.12^*^	0.73^**^	−0.32^**^	0.72^**^	–			
7. Income	2.24	0.74	–	0.25^**^	0.52^**^	0.07	0.52^**^	0.40^**^	–		
8. Followers' work engagement	3.74	0.73	0.19^**^	−0.04	0.04	−0.18^**^	0.03	−0.02	−0.08	–	
9. Subjective career success	3.15	0.90	0.15^**^	−0.04	0.02	−0.20^**^	0.02	0.02	−0.01	0.54^**^	–

** p < 0.01,

* p < 0.05;

a *0 = female, 1 = male, mean value of sex represented data of male followers*.

*The leaders' work engagement reflected group-level (work team) data*.

### The Influence of Leaders' Work Engagement on Their Followers' Subjective Career Success: Multilevel Mediation Effect of Followers' Work Engagement

Team leaders' work engagement was a group-level variable, whereas followers' work engagement and their subjective career success were individual-level variables. We followed Preacher et al. (2010, 2011) recommendations to test the 2-1-1 mediation model.

As shown in Table 2, team leaders' work engagement had a positive effect on followers' subjective career success (β = 0.192, *p* < 0.05). Followers' work engagement had a positive effect on followers' subjective career success (β = 0.994, *p* < 0.001). The results showed a significant indirect effect of team leaders' work engagement on followers' subjective career success via followers' work engagement [ab = 0.191, *p* < 0.05, 95% CI (0.019, 0.391)]. Here, we used the open-source software R to compute the 95% confidence interval by resampling the parameter estimates generated by Mplus (samples = 20,000).

**Table 2:** Multilevel mediation effect of followers' work engagement.

	***M*** **(Fworen)**		***Y*** **(SCS)**	
	**Estimate**	**SE**	**Estimate**	**SE**
**Between level**				
Intercept	2.957^***^	0.371	−0.578	0.510
*X*_b_ (Lworen)	0.192^*^	0.088	0.002	0.081
*M*_b_ (Fworen)	–	–	0.994^***^	0.177
Residual variances of *M*_b_	0.133^**^	0.039	–	–
Residual Variances of *Y*_b_	–	–	0.015	0.027
Indirect effect	0.191^*^			
Within level				
*M*_w_ (Fworen)	–	–	0.531^***^	0.071
Variances of *M*_w_	–	–	0.525^***^	0.049

* p < 0.05,

** p < 0.01,

*** p < 0.001;

*SCS, subjective career success; Lworen, leaders' work engagement; Fworen, followers' work engagement; b subscript represents between level; w subscript represents within level*.

## Discussion

### Leaders' Work Engagement and Followers' Work Engagement and Subjective Career Success

Considerable evidence about the influencing factors of subjective career success in the fields of psychology and organization science has shown that all the individual-level, group-level, and organizational-level variables have important impacts on individual subjective career success (reviews see Ng et al., 2005; Ng and Feldman, 2014; Spurk et al., 2019). Using a multilevel approach, the present study investigated the relationships among leaders' work engagement, followers' work engagement, and subjective career success. The results showed that leaders' work engagement positively affects their followers' work engagement, which affects followers' subjective career success.

The working environment is an important source of information for working group members. Various types of information, such as leaders' characteristics and working climate, will affect individuals' work attitudes and behaviors formally and informally.

Employees will influence one another in the workplace. The process by which the psychological well-being experienced by one person affects the level of well-being of another person is referred to as crossover (Bakker et al., 2009; Wirtz et al., 2017). Crossover is a dyadic, interindividual transmission of well-being between closely related individuals that occurs within a particular domain. As a positive state of mind, one's work engagement can crossover within the organization and affect other employees' work engagement, especially when the two are closely linked with the same tasks or emotions (Bakker et al., 2009). Bakker et al. (2006) examined the crossover of work engagement. The results of multilevel analyses confirmed that team-level work engagement is related to team members' work engagement after controlling for members' job demands and resources. The results of this study suggested that leaders' work engagement influenced followers' work engagement. Leaders who were vigorous, dedicated, and absorbed tended to have a positive attitude toward their work environment. They tended to be highly motivated and showed persistence in reaching their goals. As the followers observe the positive impact of their leaders' work engagement, they were more likely to emulate the behaviors associated with work engagement.

We also proposed that this effect may occur through the process of emotional contagion, thereby forming a homogenous emotional state and social cognitive state among work team members (Barsade and Gibson, 2012; Collins et al., 2013). Leaders play an important role in the formation of group emotions. Teams with a leader who shows more positive emotions are usually equipped with more enthusiasm and striving spirit. Leaders' emotions can not only influence followers in the short term but also determine the whole atmosphere of the team in the long term such that followers will be immersed in a positive or negative state of emotion for a long time. Future research can examine the role of emotional contagion in this process and test whether emotional contagion mediates the influence effect of leaders' work engagement on their followers' work engagement.

Subjective career success is considered to be an important outcome variable of work engagement and has been discussed by many studies. Some studies have indicated that work engagement is related to subjective career success, and many of them have revealed that work engagement acts as a mediator between many antecedent variables and subjective career success (Demerouti et al., 2001; Bakker et al., 2008; Giallonardo et al., 2010; Karatepe, 2012; Laschinger, 2012; Karanika-Murray et al., 2015; Jawahar and Liu, 2017). For example, Jawahar and Liu (2017) found that a proactive personality is an antecedent of job and career satisfaction, and work engagement mediates these relationships. Karanika-Murray et al. (2015) tested two models in which work engagement and its constituent dimensions (vigor, dedication, and absorption) mediated the relationship between organizational identification and job satisfaction. Karatepe (2012) revealed that work engagement fully mediated the effects of co-workers and supervisor support on career satisfaction.

As the above findings suggested, recent works on the mediating effect of engagement on subjective career success have mainly focused on individual and organizational variables. However, the present research focused on group-level variables and presented leaders' work engagement as a more distal predictor of followers' subjective career success. The results showed that leaders' work engagement influenced their followers' subjective career success by enhancing their work engagement.

### Implications

First, the results of the present research have theoretical implications for the research on organizational behavior and career success.

Although the influencing factors of subjective career success have been well-documented in the organizational behavior literature (Ng et al., 2005; Ng and Feldman, 2014; Spurk et al., 2019), the effect of work engagement on subjective career success has been highlighted in the newly emerging literature (Bakker et al., 2008; Karanika-Murray et al., 2015; Jawahar and Liu, 2017). However, less attention has been given to how the interactive relationship between leaders' and their followers' work engagement influences followers' subjective career success. The present research revealed that leaders' work engagement, as a level 2 variable, could influence followers' subjective career success, a level 1 variable, by the mediation effect of followers' work engagement. In other words, followers' work engagement functioned as a cross-level complete mediator in the relationship between leaders' work engagement and followers' work engagement. These findings extend the perspective of the earlier work from the employee level to the group level. This research deepened and expanded our understanding of the mechanism of leaders' work engagement on their followers' subjective career success.

Moreover, our results have several practical implications for organizational management and team construction.

Career success serves as an accelerator and catalyst for individual development. At the same time, it is crucial for a work team or an organization to function efficiently and progress in the long term. In this way, we should pay attention to the cultivation and development of career success in the management process. In addition, considering the positive influence of leaders' and followers' work engagement on their subjective career success, we should focus on increasing work engagement.

Leaders' work engagement can positively predict followers' subjective career success with this relationship mediated by followers' work engagement. Therefore, leaders' work engagement can be promoted and followers' work engagement can be facilitated through various approaches.

### Research Limitations and Suggestions for Future Research

First, it is important to note that the present research was conducted with participants (leaders and followers) in the Chinese cultural context. In consideration of numerous differences between oriental and occidental cultures, the results of the present research should be cautiously promoted in a cross-cultural context. Moreover, due to major differences in terms of production mode, organizational culture, and employee welfare in different organizations, we should be cautious in generalizing the results of the present research to other organizations. In addition, we adopted self-reported questionnaires as the research method. Although it was tested by Harman's one-factor model, common method variance fundamentally could not be avoided in our results. Finally, although the data for this research came from two independent questionnaires measuring team leaders and followers and controlling for possible influences of additional variables such as education background, we cannot deny its cross-sectional nature. Due to the cross-sectional nature of the study, we cannot draw any causal inferences.

Based on the limitations and problems of the current research, we offer several suggestions for future research in the following respects.

First, more research must be conducted in a cross-cultural context seeking to explore the interaction between leaders' and followers' work engagement in different cultures and to examine the applicability of the mediation effect in various cultures. Second, to improve the external validity, we should expand our sample size with samples from different organizations in different industries. Third, future work could combine self-reported evaluation with peer assessment to measure variables, reduce the impact of common method variance, and explore the relationship between variables in a more comprehensive and objective way. Fourth, future research can conduct longitudinal studies to provide more convincing evidence in the causal inference of the results. Finally, future work should compare various possible hypotheses to examine whether they could explain or to what extent they could elucidate the interaction.

## Conclusion

The results of a multilevel model demonstrated that leaders' work engagement was positively related to followers' subjective career success, with this relationship being mediated by followers' work engagement.

## Data Availability Statement

The raw data supporting the conclusions of this article will be made available by the authors, without undue reservation.

## Ethics Statement

The studies involving human participants were reviewed and approved by Research Ethics Committee of South China Normal University. The patients/participants provided their written informed consent to participate in this study.

## Author Contributions

All authors listed have made substantial, direct, and intellectual contribution to the work and approved it for publication.

## Conflict of Interest

The authors declare that the research was conducted in the absence of any commercial or financial relationships that could be construed as a potential conflict of interest.
